# Where is the family when we work with adult patients? An eating disorders perspective

**DOI:** 10.1186/2050-2974-1-S1-O5

**Published:** 2013-11-14

**Authors:** Marie-Louise Metres, Liz Wynne

**Affiliations:** 1 Eating Disorders Unit, NorthWestern Mental Health, Royal Melbourne Hospital, Australia

## 

The North Western Mental Health, Eating Disorders, Day Patient Program located at the Royal Melbourne Hospital is a 10 week program (8 weeks full time, 2 weeks part time) for adults suffering from Anorexia Nervosa, Bulimia Nervosa and Eating Disorder Not Otherwise Specified (EDNOS). The program operates 5 days a week and is designed for those who demonstrate motivation to engage in the recovery process. The program is based around group work and individuals are given the opportunity to practice eating in a variety of settings.

The service, in conjunction with CEED* has identified family work as a major component of the program. Single session work, multi-family groups and family and carer information sessions provide the foundation for this aspect of the program as they demonstrate the importance of working creatively with families and carers. These groups aim to facilitate change by; providing awareness about the lived experience of an eating disorder as well as that of a carer, endeavouring to bridge the gap between life in the community and intensive out-patient treatment and understanding the influence of family communication styles and dynamics. This presentation will highlight the importance of conducting family work in an adult setting and explore the outcome data that has been collated over the past 12 months.

* CEED is a service designed to provide clinical consultation, training, resource and service development, to build quality and sustainable eating disorder treatment responses within the public specialist mental health service.

This abstract was presented in the **Adult Treatment and Services** stream of the 2013 ANZAED Conference.

